# Role of Per1 and the mineralocorticoid receptor in the coordinate regulation of αENaC in renal cortical collecting duct cells

**DOI:** 10.3389/fphys.2013.00253

**Published:** 2013-09-17

**Authors:** Jacob Richards, Lauren A. Jeffers, Sean C. All, Kit-Yan Cheng, Michelle L. Gumz

**Affiliations:** ^1^Department of Medicine, University of FloridaGainesville, FL, USA; ^2^Department of Biochemistry and Molecular Biology, University of FloridaGainesville, FL, USA

**Keywords:** kidney, ENaC, E-box, MR, circadian clock

## Abstract

Renal function and blood pressure (BP) exhibit a circadian pattern of variation, but the molecular mechanism underlying this circadian regulation is not fully understood. We have previously shown that the circadian clock protein Per1 positively regulates the basal and aldosterone-mediated expression of the alpha subunit of the renal epithelial sodium channel (αENaC). The mechanism of this regulation has not been determined however. To further elucidate the mechanism of mineralocorticoid receptor (MR) and Per1 action, site-directed mutagenesis, DNA pull-down assays and chromatin immunoprecipitation (ChIP) methods were used to investigate the coordinate regulation of αENaC by Per1 and MR. Mutation of two circadian response E-boxes in the human αENaC promoter abolished both basal and aldosterone-mediated promoter activity. DNA pull down assays demonstrated the interaction of both MR and Per1 with the E-boxes from the αENaC promoter. These observations were corroborated by ChIP experiments showing increased occupancy of MR and Per1 on an E-box of the αENaC promoter in the presence of aldosterone. This is the first report of an aldosterone-mediated increase in Per1 on a target gene promoter. Taken together, these results demonstrate the novel finding that Per1 and MR mediate the aldosterone response of αENaC through DNA/protein interaction in renal collecting duct cells.

## Introduction

The circadian clock regulates the rhythmic fluctuation of physiological processes, including but not limited to: immune, reproductive, vascular, endocrine, blood pressure (BP), and renal function (Lowrey and Takahashi, 2004; Agarwal, 2010; Stow and Gumz, 2011; Richards and Gumz, 2012). The mammalian clock can be divided into two components: the central circadian clock located in the suprachiasmatic nuclei in the hypothalamus of the brain, which synchronizes itself in response to light, and the peripheral clocks that exist in almost every organ and tissue. The entrainment of the peripheral clock occurs via mechanisms that are thought to act both independently and dependently of the central clock (Dibner et al., 2010; Richards and Gumz, 2012). At the molecular level, the circadian clock mechanism is regulated by a transcription and translation oscillating loop, which consists of four core circadian proteins. The heterodimer of the transcription factors circadian locomotor output cycles kaput (CLOCK) and brain and muscle ARNT (aryl hydrocarbon receptor nuclear translocator)-like 1 (BMAL1) stimulate gene transcription by binding to response elements (E-boxes) present in the clock-controlled gene promoters. Among the genes activated by CLOCK and BMAL1 are their own repressors encoded in the Period (*Per1, Per2*, and *Per3*) and Cryptochrome (*Cry1* and *Cry2*) genes (Albrecht and Eichele, 2003). In each peripheral organ, the circadian clock drives rhythmic expression of thousands of genes through interaction with the E-box response elements. Recent evidence suggests novel mechanisms of circadian regulation including the interaction of the circadian clock proteins with nuclear receptors and the existence of co-regulatory mechanisms (Lamia et al., 2011) [reviewed in Richards and Gumz (2013)]. Profiling experiments demonstrated that a multitude of nuclear receptors were shown to exhibit rhythmic oscillations in adipose, liver, and muscle tissue (Yang et al., 2006).

Aldosterone is a mineralocorticoid steroid hormone involved in regulation of sodium reabsorption and BP control. Aldosterone action is primarily mediated via the mineralocorticoid receptor (MR). Plasma aldosterone levels fluctuate with a circadian pattern in humans and mice (Agarwal, 2010; Nikolaeva et al., 2012). The molecular connection between aldosterone action and the circadian clock remains largely unknown. However, previous work from our lab demonstrated that the circadian protein Per1 is an early aldosterone target (Gumz et al., 2003).

αENaC is the regulated subunit of the renal epithelial sodium channel (ENaC) (Palmer et al., 2012). The circadian protein Per1 positively regulates the basal transcription and the aldosterone-induction of the *Scnn1a* (hereafter referred to as αENaC) gene (Gumz et al., 2009, 2010). This regulation occurs through interactions with an E-box element located in the promoter. Pharmacological blockade of Per1 translocation into the nucleus prevents Per1 from interacting with promoter E-box resulting in reduced basal level and aldosterone-mediated induction of αENaC, and decreased ENaC activity (Richards et al., 2012). Per1 also coordinately regulates multiple other genes involved in sodium reabsorption in the kidney (Stow et al., 2012). This regulation includes the positive regulation of Fxyd5, a positive regulator of the Na,K-ATPase (Lubarski et al., 2005), and the negative regulation of Endothelin-1 and Caveolin-1. Endothelin-1 is a potent inhibitor of ENaC channel activity through both the Endothelin-A and Endothelin-B receptors via a nitric-oxide dependent mechanism (Bugaj et al., 2008; Lynch et al., 2013). Caveolin-1 is a lipid raft protein, which retrieves ENaC from the membrane (Lee et al., 2009). The regulation of these genes by Per1 predicts that loss of Per1 should result in renal sodium wasting, decreased plasma volume, and subsequent decreased BP. Indeed, we have shown that Per1 KO mice have lower BP compared to wild type (WT) mice (Stow et al., 2012).

Since Per1 regulates the basal and the aldosterone-mediated regulation of αENaC (Gumz et al., 2009, 2010; Richards et al., 2012), we hypothesized that Per1 and MR may act coordinately on αENaC expression during the aldosterone response. Here we report the presence of Per1 and MR at the E-box response elements from the αENaC promoter in the renal cortical collecting duct cell line mpkCCD_c14_. Mutations of the E-boxes in the human promoter abolished both basal and aldosterone-mediated promoter activity. DNA pull down assays demonstrated the interaction of both MR and Per1 with a specific E-box from the promoter. These interactions were confirmed on the endogenous αENaC promoter using chromatin immunoprecipitation (ChIP). Taken together, these results demonstrate coordinate regulation of αENaC expression by Per1 and MR during the aldosterone response and demonstrate a potential mechanism for αENaC gene regulation by MR and a circadian clock protein.

## Materials and methods

### Cell culture and hormone treatment

The mpkCCD_c14_ cells were a gift from Dr. Alain Vandewalle (INSERM, Paris, France) (Bens et al., 1999). All cells were maintained in DMEM/F-12 (Invitrogen) plus 10% fetal bovine serum (FBS) and 50 μg/ml gentamicin (Sigma).

### Construction of E-box mutations in the αENaC promoter

Mutations of the αENaC promoter-luciferase construct were made using QuikChange® Site-Directed Mutagenesis Kit (Stratagene) according to the manufacturer's instructions. Specific primers were used to mutate putative E-boxes in the αENaC promoter, creating new restriction sites that were verified by both restriction enzyme digests and DNA sequencing (Table 1). The human promoter was analyzed for putative E-box motifs using TF Search (http://www.cbrc.jp/research/db/TFSEARCH.html) as described (Gumz et al., 2010).

**Table 1:** **Mutation of E-boxes in αENaC promoter-luciferase construct**.

**Plasmid**	**mE-box 1**	**mE-box 2**
E-box sequence	ATCCAGCTGTCC	CTTCACCTGGGC
Mutated sequence	ATCCAGCTAGCC	CGGTACCTGGGC
Forward primer	5′CAATGAAGAAAAATCCAGCTAGCCCTTCCAAGGGGAGGTATC	5′CGCCTAGCCCCCAGCGGTACCTGGGCCCCTCCC
Reverse primer	5′GATACCTCCCCTTGGAAGGGCTAGCTGGATTTTTCTTCATTG	5′GGGAGGGGCCCAGGTACCGCTGGGGGCTAGGCG
New restriction digest site	*Nhe1*	*Kpn1*

### Luciferase assays

Approximately 192,000 mpkCCD_c14_ cells were seeded in 24-well plates (Corning). Twenty-four hours later cells were transfected with pGL3 (Promega), a human αENaC promoter-luciferase construct (gift of Dr. Christie Thomas, University of Iowa), or a mutated promoter-luciferase construct. Transfections were performed using lipofectamine (Invitrogen), according to the manufacturer's instructions, in serum-depleted media. 1 μ M Aldosterone or vehicle (ethanol) treatment was administered 24 h later. Final ethanol concentration in both vehicle and aldosterone treated cells was 0.1%. All cells were co-transfected with equal amounts of the plasmid pRL-TK (Promega). Transfection efficiency was normalized to *Renilla* luciferase levels. Dual-luciferase assays (Promega) were performed according to the manufacturer's instructions.

### Nuclear extracts, DNA affinity purification assays (DAPA), and immunoblotting

Nuclear and cytosolic extracts were isolated using the NE-PER kit (Pierce) according to the manufacturer's instructions. For DNA affinity purification assay (DAPA), Probes were immobilized on 50 μ l of streptavidin-coated agarose beads (Sigma) and incubated with 175 μ g of nuclear mpkCCD_c14_ extracts either treated with vehicle (ethanol) or 1 μ M aldosterone for 24 h in the presence of freshly prepared protease inhibitors (Fischer) for 2 h at 4°C with end-over-end rotation. Beads were pelleted. Supernatants were removed and used for input controls by Western blotting for actin. Pelleted beads were washed four times with ice-cold PBS plus protease inhibitors. After the final wash, all liquid was aspirated from the beads with flat-headed gel loading tips. Then 50 μ l of Laemmli sample buffer (Invitrogen) plus β-mercaptoethanol was added and samples were boiled for 5 min, chilled on ice, and loaded onto a BioRad 4–20% Mini-PROTEAN® TGX™ Precast Gel for electrophoresis. Samples were then transferred to a polyvinylidene fluoride (PVDF) membrane. The membrane was blocked in 2% non-fat dry milk in TBS-S [Tris-buffered saline (TBS) plus 0.05% Rodeo™ Saddle Soap] (USB) and incubated overnight at 4°C with anti-CLOCK (1:1000) (Pierce), anti-Per1 (1:500) (Pierce), anti-rMR1-18 1D5 (anti-MR) (1:500) (DSHB), or anti-actin (1:500) (Santa Cruz) antibodies. The rMR1-18 1D5 developed by Dr. Gomez-Sanchez was obtained from the Developmental Studies Hybridoma Bank developed under the auspices of the NICHD and maintained by the University of Iowa, Department of Biology, Iowa City, IA 52242. The membrane was washed with 2% non-fat dry milk in TBS-S for 15 min and then incubated with horseradish peroxidase conjugate anti-rabbit secondary antibody or anti-mouse secondary antibody (for anti-MR) and incubated in 2% non-fat dry milk in TBS-S for 1 h at 4°C. After incubation, the blot was washed with TBS-S for 15 min. Detection was performed using Novex® ECL Chemiluminescent Substrate reagents (Invitrogen). The sequences of the DAPA probes were (E-box sequence is underlined): wild-type human E-box 1 (5′CAATGAAGAAAAATCCAGCTGTCCCTTCCAAGGGGA), mutated -human E-box 1 (5′CAATGAAGAAAAATCCAGCTAGCCCTTCCAAGGGGA), wild-type human E-box 2 (5′CCTAGCCCCCAGCTTCACCTGGGCCCCTCCCGGGTC), and mutated human E-box 2 (5′CCTAGCCCCCAGCGGTACCTGGGCCCCTCCCGGGTC).

### Chromatin immunoprecipitation (ChIP)

The mpkCCD_c14_ cells were grown to ~80% confluency and then treated with vehicle (ethanol) or 1 μ M aldosterone for 24 h. ChIP was performed using the ChIP-IT^tm^ Express Enzymatic Kit (Active Motif) according to the manufacturer's instructions. Chromatin concentrations were calculated and equal amounts of vehicle-treated and 1 μ M aldosterone chromatin were used per pull down. Pull downs were performed using 3 μg of either anti-Per1 (Pierce), anti-rMR1-18 1D5 (anti-MR) (DSHB, Iowa), anti-Pol II (Santa Cruz), or rabbit IgG (Bethyl) and were incubated overnight at 4°C with end-over-end rotation. Immunoprecipitated DNA was amplified by End Point PCR with primer pairs that flanked the previously identified Per1 binding E-box (Gumz et al., 2010). (Forward 5′ATTCCTGGCCTATCAGCCAA) (Reverse 5′AAAGAGAATGGGTCCCCCAA). Band intensities were quantitated using densitometry, which was performed using ImageJ (rsbweb.nih.gov/ij). Bands were relativized to the relevant vehicle or aldosterone-treated 10% input.

### Statistics

All experiments, unless otherwise stated, were performed in duplicate in at least three independent studies. Two-tailed student's unpaired *t*-test (Microsoft Excel) was used to test statistical significance and *p* < 0.05 was considered significant. Data are presented as the means ± S.E.

## Results

### E-box response elements in the αENaC promoter contribute to aldosterone response

**Figure 1:**
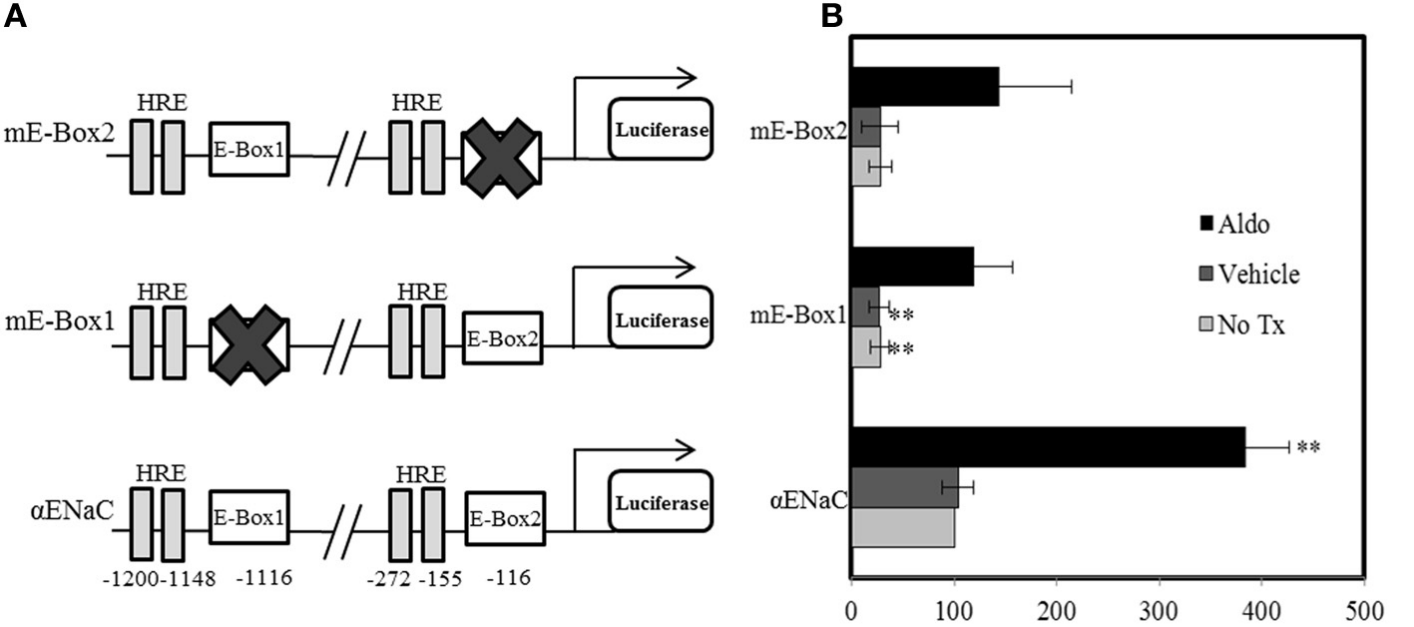
**Mutation of E-box elements inhibits basal and aldosterone-mediated αENaC promoter activity. (A)** Cartoon of the αENaC promoter indicating E-box sites that were mutated and nearby hormone response elements (HRE) (not to scale). The position of each E-box element and HRE relative to the transcription start site is indicated. **(B)** Cells were transfected with the pRL renilla luciferase and a plasmid containing the αENaC promoter or a mutated form, cloned upstream of the firefly luciferase cDNA. E-box 1 (TCCAGCTGTC) at −1116, relative to the transcription start site was mutated to mE-box 1 (TCCAGCT**AG**C) and E-box 2 (TTCACCTGGG) at −116 was mutated to mE-box 2 (**GGT**ACCTGGG). Cells were either not treated (No Tx) or treated with vehicle or aldosterone (aldo) for 24 h. Data are presented as the mean ± standard error, *n* = 6, ^**^p < 0.01 vs. α ENaC/luc + no treatment.

Circadian clock proteins mediate their effects on gene expression via binding to E-box response elements in the promoters of target genes. Per1 does not contain a DNA binding domain, so it likely binds target sites in DNA by forming a complex with a binding partner. Per1 and CLOCK were both detected at an E-box from the mouse αENaC promoter (Gumz et al., 2010). Promoter analysis of the human promoter was conducted using TF Search to look for E-box sequences in close proximity to hormone response elements (HREs). Two such sites were identified, E-box 1 and E-box 2, located at positions −1116 and −116, respectively, relative to the transcription start site (Figure 1A). To generate human αENaC promoter constructs with defective E-boxes, mutations were constructed at both sites. Mutated sequences were checked with TF search to confirm disruption of the consensus site. mpkCCD_c14_ cells were transfected with the wild-type αENaC promoter-luciferase construct, the mutant mE-box 2 reporter vector, or the mutant mE-box 1 plasmid. Twenty-four hours later, cells were treated with vehicle or aldosterone for 24 h. Mutation of either E-box element led to an approximate 75% overall decrease in luciferase activity, indicating reduced promoter function in the absence of either E-box (Figure 1B). The decreases were evident in both basal and aldosterone-induced promoter activity.

### Per1 and MR interact with E-box response elements from the human αENaC promoter in an aldosterone-dependent manner

**Figure 2:**
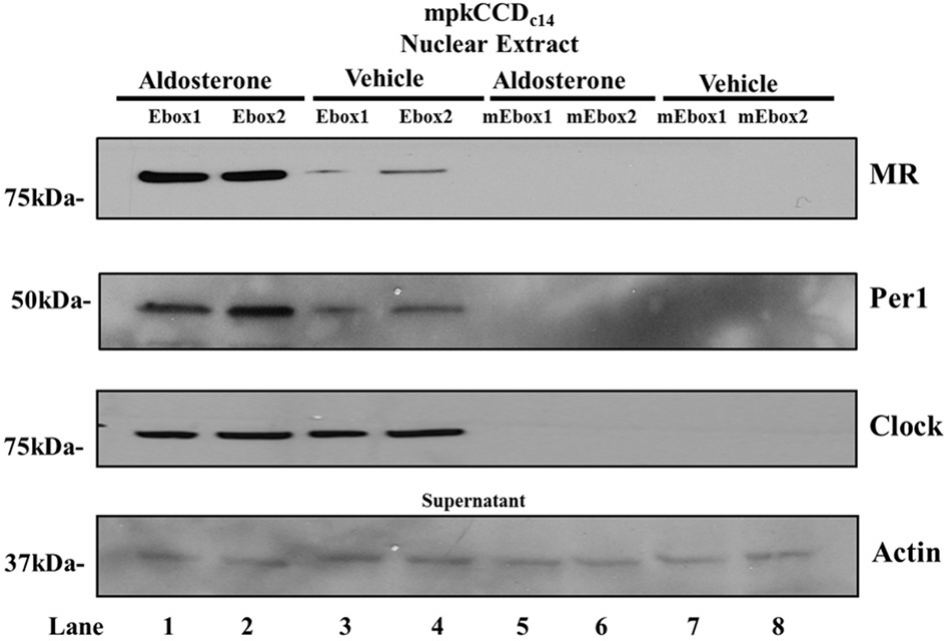
**Per1 and MR interact with E-boxes from the αENaC promoter**. Nuclear extracts from mpkCCD_c14_ cells treated with vehicle or aldosterone were incubated with biotinylated probes from the human wild-type (Lane 1–4) or mutated (Lane 5–8) E-box 1 (−1116) and human E-box 2 (−116) to perform DAPA. Western blot analysis was performed using anti-MR, anti-Per1 or anti-Clock. anti-Actin was used as a loading control on supernatants. Data are representative of 3 independent experiments. mE-box 1 and mE-box 2 represent mutated E-box probes used as a negative control. Mutations made to these sequences exactly match the E-box mutations made in Figure 1.

To further investigate Per1 and aldosterone-mediated regulation of αENaC, a DAPA was performed. We hypothesized that if the E-boxes in the αENaC promoter were required for aldosterone action, MR may interact with these elements. 5′ biotinylated oligonucleuotide probes representing wild-type and mutated human E-box 1 and E-box 2 were incubated with nuclear extracts from mpkCCD_c14_ cells treated for 24 h with either vehicle or aldosterone. MR was found to complex with the E-box response elements in an aldosterone-dependent manner (Figure 2, Lanes 1–4). Interaction of Per1 increased at both E-boxes in aldosterone-treated cells, supporting the hypothesis that these sites represent aldosterone-responsive circadian response elements. CLOCK was found to bind to both E-boxes but was not significantly increased under these conditions in the presence of aldosterone. Importantly, interaction of Per1, MR, and CLOCK with E-box 1 and E-box 2 was abolished upon mutation of the binding site (Figure 2, Lanes 5–8). Thus, the interaction of MR and Per1 with the E-box response elements from the human αENaC promoter appears to be aldosterone-dependent and sequence specific.

### Aldosterone leads to increased occupancy of per1 and MR on an E-box in the αENaC promoter in mpKCCD_c14_ cells

**Figure 3:**
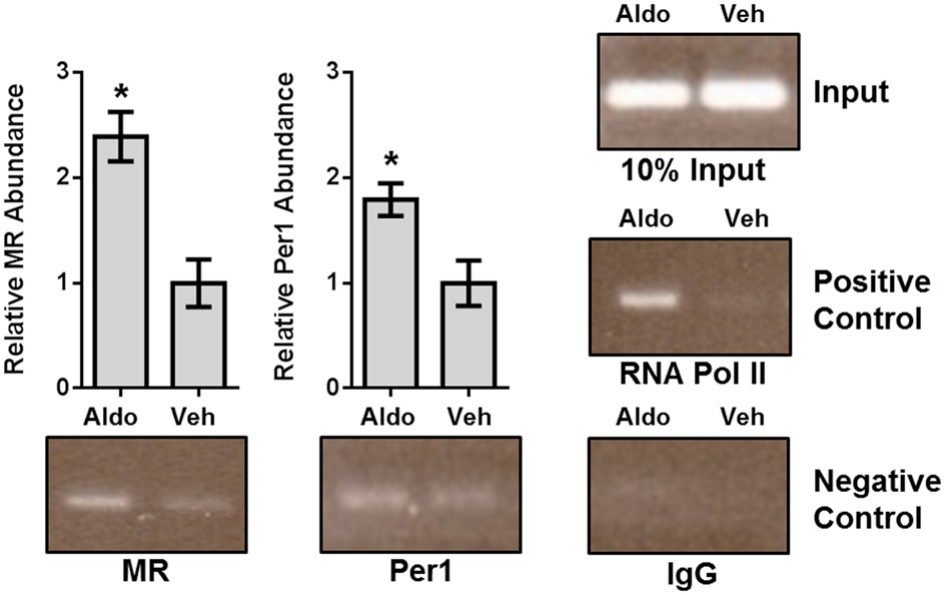
**Aldosterone treatment leads to increased occupancy of Per1 and MR on the αENaC promoter in mpkCCD_c14_ cells**. Chromatin immunoprecipitation experiments were performed using mpkCCD_c14_ cells treated with either vehicle (ethanol) or 1 μ M aldosterone for 24 h. Chromatin immuprecipitations were performed using anti-Per1 (Pierce), anti-rMR1-18 1D5 (anti-MR) (DSHB), anti-Pol II (Santa Cruz), or rabbit IgG (Bethyl) (negative control) antibodies. Endpoint PCR was performed using primers flanking the previously determined E-box in the mouse αENaC promoter. Bands were quantitated using densitometry, which was performed using ImageJ (rsbweb.nih.gov/ij). Signal strength was normalized to the relevant vehicle or aldosterone treated input control. *N* = 3 for MR, Per1, and IgG, *n* = 2 for RNA pol. Values are represented as the mean ± SEM. ^*^p < 0.05, Aldosterone vs. Vehicle.

To further corroborate our *in vitro* findings of the aldosterone-dependent interactions of Per1 and MR on the E-box response elements, ChIP experiments were conducted using mpkCCD_c14_ cells treated with vehicle or aldosterone for 24 h (Figure 3). Aldosterone resulted in increased occupancy of RNA polymerase II on this region of the αENaC promoter, consistent with increased transcription of the gene. Importantly, aldosterone treatment resulted in increased MR and Per1 occupancy, consistent with the *in vitro* DNA pull down experiments in Figure 2. These ChIP results provide the first direct evidence for the presence of Per1 and MR in a region of the endogenous αENaC promoter that includes an E-box in response to aldosterone.

## Discussion

Here we provide substantive mechanistic evidence for co-regulation of the αENaC gene by Per1 and MR. The two transcription factors activate in an aldosterone-dependent manner. Promoter-luciferase assays, DAPA, and ChIP consistently demonstrated a role for Per1 and E-box response elements in the aldosterone-mediated regulation of αENaC. For the first time it was shown that MR and Per1 both interact with canonical E-box circadian response elements located within the 5′ regulatory region of the human αENaC promoter. ChIP analysis also demonstrated that MR and Per1 are both present on a region of the endogenous mouse αENaC promoter containing a canonical E-box, providing the first direct evidence of Per1 occupancy on the αENaC promoter.

It is important to note that a putative HRE is located within the ChIP amplicon and in close proximity to the E-box (−770 for the HRE, −689 for the E-box). As shown above (Figure 1A), several HREs are located within close proximity to the E-boxes in the human αENaC promoter. Because the E-boxes and apparent HREs are so close together, ChIP alone does not allow unambiguous resolution of the MR binding site in this region. However, evidence from the DAPA experiments supports a model in which MR and Per1 interact with the E-box response element of the αENaC gene promoter. The E-boxes appear to be critical for the aldosterone induction of αENaC in collecting duct cells.

It is likely that Per1 is associating with other components of the canonical clock complex such as CLOCK and BMAL1 as the Per1 protein does not contain an inherent DNA binding domain (Kucera et al., 2012). In this study, we demonstrate CLOCK and Per1 binding to the same E-boxes in our DAPA experiments. However, further experiments are needed to clarify the exact mechanism of this interaction and to identify the specific proteins Per1 associates with in order to interact with the E-box response elements in the αENaC promoter.

E-boxes have previously been implicated as transcriptional targets for glucocorticoid action (Singletary et al., 2008). MR is highly homologous to glucocorticoid receptor (GR) and both receptors are ligand-dependent transcription factors (Arriza et al., 1987; Kohn et al., 2012). MR and GR share 94% primary sequence homology in the DNA binding domain, and both receptors share the same HREs in several genes, including αENaC (Arriza et al., 1987; Chen, 1999; Mick et al., 2001). Both nuclear receptors contribute to the aldosterone-mediated induction of the Per1 gene (Gumz et al., 2003, 2009). This result is consistent with previous findings that both Per1 and Per2 contribute to coordinate circadian control of other metabolic pathways in peripheral tissues via nuclear receptor signaling pathways (Albrecht et al., 2001; Schmutz et al., 2010). Lamia et al. have shown that other circadian clock proteins, Cry1 and Cry2, can interact with the GR, bind to the glucocorticoid response element in the phosphoenolpyruvate carboxykinase 1 promoter, and subsequently repress GR action (Lamia et al., 2011). These earlier studies provided precedent for coordinate action of MR and Per1 on transcriptional regulation of αENaC.

The circadian clock plays an important role in the control of BP and renal function (Richards and Gumz, 2013). CLOCK KO mice have lower BP, dysregulated sodium excretion (Zuber et al., 2009) and the loss of circadian expression of plasma aldosterone levels (Nikolaeva et al., 2012). BMAL1 KO mice exhibit reduced BP during the active phase (Curtis et al., 2007). Cry1/Cry2 KO mice exhibit salt sensitive hypertension due to an up-regulation in the aldosterone synthesis enzyme 3-β-dehydrogenase-isomerase leading to increased aldosterone synthesis and high aldosterone levels (Doi et al., 2010). Both the CLOCK KO and Cry1/Cry2 KO phenotypes and their dysregulated aldosterone levels provide additional evidence of a connection between the circadian clock and aldosterone signaling. Together with our finding that Per1 is an early aldosterone target (Gumz et al., 2003), the present study demonstrates that MR and Per1 interact with E-boxes in the αENaC promoter. These data provide additional evidence for the role of the circadian clock in aldosterone signaling. The coordinated action of MR and Per1 may suggest a previously unrecognized mechanism by which the circadian clock modulates physiological rhythms and aldosterone signaling.

### Conflict of interest statement

The authors declare that the research was conducted in the absence of any commercial or financial relationships that could be construed as a potential conflict of interest.
